# Determinants of child and forced marriage in Morocco: stakeholder perspectives on health, policies and human rights

**DOI:** 10.1186/1472-698X-13-43

**Published:** 2013-10-16

**Authors:** Alexia Sabbe, Halima Oulami, Wahiba Zekraoui, Halima Hikmat, Marleen Temmerman, Els Leye

**Affiliations:** 1 ICRH - International Centre for Reproductive Health, Faculty of Medicine & Health Sciences, Ghent University, De Pintelaan 185 UZP114, 9000 Ghent, Belgium; 2 AEDF - Association El Amane pour le Développement de la Femme, Avenue Ben Noussair, Sidi Youssef Ben Ali, Marrakech, Morocco; 3 WHO - World Health Organization, Geneva, Switzerland; 4 Faculty of Medicine and Health Sciences, Ghent University, Ghent, Belgium

**Keywords:** Child and forced marriage, Morocco, Women’s rights, Sexual and reproductive health, Violence

## Abstract

**Background:**

In Morocco, the social and legal framework surrounding sexual and reproductive health has transformed greatly in the past decade, especially with the introduction of the new Family Law or Moudawana. Yet, despite raising the minimum age of marriage for girls and stipulating equal rights in the family, child and forced marriage is widespread. The objective of this research study was to explore perspectives of a broad range of professionals on factors that contribute to the occurrence of child and forced marriage in Morocco.

**Methods:**

A qualitative approach was used to generate both primary and secondary data for the analysis. Primary data consist of individual semi-structured interviews that were conducted with 22 professionals from various sectors: health, legal, education, NGO’s and government. Sources of secondary data include academic papers, government and NGO reports, various legal documents and media reports. Data were analyzed using thematic qualitative analysis.

**Results:**

Four major themes arose from the data, indicating that the following elements contribute to child and forced marriage: (1) the legal and social divergence in conceptualizing forced and child marriage; (2) the impact of legislation; (3) the role of education; and (4) the economic factor. Emphasis was especially placed on the new Family Code or Moudawana as having the greatest influence on advancement of women's rights in the sphere of marriage. However, participants pointed out that embedded patriarchal attitudes and behaviours limit its effectiveness.

**Conclusion:**

The study provided a comprehensive understanding of the factors that compound the problem of child and forced marriage in Morocco. From the viewpoint of professionals, who are closely involved in tackling the issue, policy measures and the law have the greatest potential to bring child and forced marriage to a halt. However, the implementation of new legal tools is facing barriers and resistance. Additionally, the legal and policy framework should go hand in hand with both education and increased economic opportunities. Education and awareness-raising of all ages is considered essential, seeing that parents and the extended family play a huge role in marrying off girls and young women.

## Background

In the last decade, the social and legal framework surrounding sexual and reproductive health in Morocco has seen a profound transformation. The reform of the Family Code or Moudawana in 2004 endows Moroccan women with groundbreaking rights that were previously unheard of. Its predecessor was based on conservative Islamic family law traditions, which included provisions such as the wife’s obedience to her husband and the husband’s right of repudiation [1,2]. The new Moudawana, however, stipulates that spouses have equal rights and duties in the family, establishing a right to divorce for women, eliminating the requirement for women to have the consent of a marital guardian *(wali)* to marry and granting women more rights in the negotiation of marriage contracts [3]^a^. Also, in an attempt to bring the occurrence of child and forced marriages to a halt, the minimum age of marriage for girls was raised from 15 to 18 [4]^b^. Nevertheless, in the years following the adoption of the Moudawana, questions were raised by several NGO’s and human rights groups concerning its application, especially in the area of child marriage. Although the minimum age for girls was increased, the new Family Code does present the opportunity to ask a judge to authorize marriage before the age of 18^c^. Few of the applicants are male^d^. This loophole is increasingly being used to marry off girls, often as young as 15.

Effectively, with the new Family Code, child marriage was placed under control of the magistrates. In order to grant a child marriage, the law requires not only a medical and psychological exam of the child, but also a social inquiry into the reasons for marriage [5]^e^. Yet, despite the fact that underage marriage should remain an exception, it seems to almost have become the rule.

The rates of child marriage have risen steadily in the years following the introduction of the new Moudawana. In 2007, 33.596 underage marriages were allowed to take place [6]. Figures released by the Ministry of Justice for the year 2010 reveal that 41.098 child marriages were authorized. Compared to 2009, this represents a 23,59% increase [7]. With the judiciary approving over 90% of petitions, the practice of child marriage in Morocco is effectively upheld^f^.

Forced marriage is recognised as a human rights abuse, violating a number of international human rights norms, including the rights to freely enter into marriage, and to bodily and sexual integrity. As early as 1948, the right to free and full consent to a marriage was stipulated by the Universal Declaration of Human Rights^g^, acknowledging that consent cannot be free and full when one of the parties is not sufficiently mature to make an informed decision, as is the case with child marriage [8]. The most widely ratified United Nations Convention on the Rights of the Child (CRC) defines a child as anyone under the age of 18 years^h^, based on ideas of universalised notions of maturity [9]. Child marriage is explicitly mentioned in a number of international human rights instruments, most notably in the Convention on the Elimination of All Forms of Discrimination against Women (CEDAW), which states that the “betrothal and the marriage of a child shall have no legal effect”^i^. However, many countries legally allow young people between the ages of 16–18 to marry with parental consent, which raises questions regarding the concepts of childhood and marriage across cultures [10]. This brings to light the difficulties in defining forced marriage and child marriage. While child marriage is generally included in the scope of forced marriage [9,11], it should be noted that the primary concern lies in the incidences of forced child marriage, where explicit or implicit pressure is exerted. Research shows that young people under 18 are at a higher risk of forced marriage [12]. Most of these marriages are set up by parents and girls rarely meet or get to know their future husband before the wedding [13]. In a forced marriage, at least one of both parties is coerced into a marriage against their will and under duress. In contrast, an arranged marriage is characterized by the fact that parents or relatives introduce the spouses but both parties give their full and free consent to the union. Therein lies the difference with forced marriage [14]. However, in practice it can become difficult to accurately determine at what point emotional pressure becomes great enough to implicate genuine force in order to distinguish between forced and arranged marriages [15,16].

The overall prevalence of forced marriages is difficult to estimate, as victims rarely come forward. The practice is mostly hidden and incidences of forced marriage go underreported [17]. Figures for child marriage are easier to come by, seeing that the spouses’ ages at the date of marriage, allows for the numbers of child marriages to be quantified. This offers an objective benchmark to assess the evolution with regards to girl’s and young women’s rights.

Forced marriage and child marriage have considerable detrimental health and social consequences. These unions hinder educational development and limit opportunities, seriously affecting their economic status. Quite often it exposes them to a lifetime of domestic violence and abuse as they lack standing and power within their households [18]. Several studies confirm wide age gaps between younger married women and their spouses. This age gap clearly creates unequal power relations between the young bride and her older and more experienced husband, resulting in husbands having total control over sexual relations and decision-making [18]. Since most brides are socially conditioned not to question the authority of their husbands, they are often unable to use contraception or to plan their families. The combined effect of these factors may also make brides more likely to tolerate partner violence and not leave abusive husbands [19].

Forced marriage and child marriage in particular bring about a wide range of health consequences, of which girls and young women bear the greatest burden. Forced sexual intercourse can lead to gynaecological problems. Associations have been found between coerced first sexual intercourse and genital tract symptoms [20]. Studies also report significant associations between sexual abuse and sexually transmitted infections, bacterial vaginosis, abnormal vaginal discharge, and psychological and mental disorders [21]. Due to their bodies being not fully developed, mothers under the age of 18 years experience higher rates of maternal mortality and higher risk of obstructed labour, postpartum haemorrhage and sepsis [13,22,23]. Pregnancy related deaths are among the leading causes of mortality for 15–19 year old girls worldwide [24]. Those who give birth under the age of 15 are five times more likely to die as compared to women in their early 20s [25]. Young women and girls forced into marriage are additionally exposed to a greater risk of HIV infection [22]. Research suggests that in some settings 15- to 19-year old married girls have higher rates of HIV infection than their sexually active unmarried peers [26-28]. However counterintuitive this seems, Clark et al. [28] suggest that married girls in any setting may be more vulnerable to HIV infection because, in trying to prove their fertility, girls have high-frequency, unprotected sexual intercourse with their spouse. The husbands are often much older men, which increases their chances of having contracted HIV through previous sexual partnerships or polygamous unions [13,28].

Considering the levels of physical and psychological abuse frequently associated with forced marriage, the issue is perceived as a form of violence against women [29]. Recent figures from a study conducted by the Moroccan High Commission for Planning (HCP) reveal that women who married without consent are almost three times more likely to experience partner violence, including sexual violence, in the domestic household. Moreover, younger married women, from 18–24 years, experience a higher rate of violence than married women between 35–39 years [30]. Effects of violence during pregnancy include delayed prenatal care, sexually transmitted infections (STIs), vaginal and cervical infections, kidney infections, miscarriages and abortions, premature labour and foetal distress [31]. Another study conducted by a national network of counselling centres in Morocco indicates that minor girls are increasingly experiencing gender-based violence, of which sexual violence figures most prominently at 28%, compared to physical violence in 21% of the cases [32].

Concerns are rising in Morocco over these rates of violence against women and the swelling numbers of child marriages. The goal of this research study is to explore perspectives of a wide range of professionals on factors that contribute to the occurrence of child and forced marriage in Morocco.

## Methods

### Research design

A qualitative approach was used to generate both primary and secondary data for the analysis. The primary data entailed the use of in-depth interviews, whereas sources of secondary data included academic papers, government and NGO reports, various legal documents (new Family Code, Penal Code and the new Constitution) and media reports.

Individual semi-structured interviews were conducted with stakeholders who come into contact or deal with child and forced marriage, among which health care workers, legal professionals, teachers/academics, NGO and government representatives.

A snowball sampling strategy was used. Recruitment was focused on key-persons and key-organisations specialized in violence against women, and forced and child marriage. First-wave participants were suggested by our local contact, the law department of the University Mohammed V in Rabat. In turn, these first-wave participants indicated other potential participants. Stakeholders were recruited in 3 distinct areas in Morocco (Rabat, Casablanca and the Marrakech region). These areas were chosen as they harbour a large concentration of professionals working on the issue, and multiple (national) NGO’s and women’s rights networks in Morocco. Additionally, a number of stakeholders have experience of working in urban and rural areas throughout the entire country.

Interviews were conducted with 22 of the 41 stakeholders that were approached who have firsthand experience of forced and child marriage. Research by Guest et al. demonstrated that saturation, the point at which no new information or themes are observed in the data, occurred within the first twelve interviews [33]. A sample size of 20–30 interviews should, therefore, suffice when the aim is to understand perceptions and experiences among a group of stakeholders working on the same common theme.

Within the group of participants, there were 3 teachers/academics, 5 health care workers, 3 lawyers, 3 government representatives and 8 NGO representatives. Interviews were carried out between March 2011 and March 2012.

A semi-structured interview schedule, consisting of open-ended questions, was used. Questions explored participants’ knowledge, experience and views on forced marriage, including current policies, guidelines and recommendations to improve tackling the issue. Participants were also asked to identify obstacles in preventing child and forced marriage, and barriers to meeting the needs of girls and young women. Ideas expressed in the secondary data and in earlier interviews, were discussed in subsequent interviews.

Interviews were held in French or Arabic. Audio recordings were transcribed and transcripts of Arabic interviews were translated into English.

The purpose of the study and the terms regarding recording of the interview and anonymity were explained at the beginning of each interview before obtaining the participants’ consent. This study received ethical approval from the Ethics Board within the Faculty of Medicine & Health Sciences of Ghent University; and was exempted from ethical approval by the Comité d’Ethique pour la Recherche Biomédicale of the Faculty of Medicine and Pharmacy of Université Mohammed V in Rabat, Morocco.

### Data analysis

Thematic qualitative analysis was conducted, in which patterns or themes within the data are analysed and reported using a thematic framework [34]^j^. The framework approach allowed comparison by theme [35]. Two authors independently scrutinized transcriptions, field notes and documents, and common themes amongst the data were identified. Extracts of data from the interviews (primary data) and literature (secondary data) were categorised under the initially developed themes.

These two authors compared notes, reconciled any divergence and independently reviewed the transcripts. Findings were consecutively discussed between all first-hand data collectors.

As participants’ verbatim comments are used, they have been anonymised to protect participants’ identities. Participants’ quotes are presented with the research ID assigned during the study (f.ex. Participant 1, 2, etc.).

## Results

Four major themes arose from the data: (1) the legal and social divergence in conceptualizing forced and child marriage; (2) the impact of legislation; (3) the role of education; and (4) the economic factor.

### Legal and social divergence in conceptualizing forced and child marriage

Participants were very vocal in condemning forced and child marriage, comparing it to rape and remarking on its pervasive nature across age and social class.

*“This type of marriage is child rape; that is to say, her life has been raped by means of this marriage*.*”* (Participant 16 – health care worker)

*“There are people from disadvantaged sectors, Berbers, whose ages do not exceed 16 and with an exceedingly low level of education, just as there are wealthy, highly educated families, whose marriage age sometimes exceeds 26 and who are still subjected to this type of treatment. .... Sometimes we find that even the family has become a victim of coercion, and we are obliged to support both the girl and her family together.”* (Participant 22 – lawyer)

Yet, from a legal vantage point, academic and legal participants drew attention to the “impossibility” of a forced marriage. In accordance with the law, the marriage contract must always be signed by both spouses, so, consistent with prevailing laws, forced marriage as such could not exist.

*There is pressure, often from the family, that is a given. But once there is a signature, suggesting an agreement to the marriage, the contract is valid. Without consent the contract is void, and therefore the marriage has never taken place.* (Participant 3 – academic)

This highlights the difficulties in conceptualizing a forced marriage^k^. Depending on the circumstances, it is difficult to determine whether or not the union was entered into freely. It is clear-cut in the case of physical violence actually denying a person’s freedom of consent. Yet, feelings of anxiety and fear can overrule any resistance to a marriage, leaving a person vulnerable and unable to escape the union.

*“When women and girls are approached, they either deny or often don’t respond to the question if the marriage was forced. In light of prevailing law on marriage, consent is fundamental, so it’s problematic to regard the union as forced in these cases.”* (Participant 2 – academic)

In the case of child marriage, the lack of maturity makes consent impossible. For that reason, authorizing an underage marriage should remain exceptional and treated with delicacy. As pointed out in the introduction, official figures tell a whole different story: child marriages authorized in family courts are increasing. To justify or explain this trend, a few participants referred to situations in which judges want to protect young girls from stigma, or provide the opportunity for a “better” life abroad.

*“When faced with social taboos, such as loss of virginity or pregnancy, judges usually grant authorization to go through with the marriage. Otherwise it would be extremely detrimental to the girl’s status”* (Participant 14 – government representative)

*“Sometimes the girls standing in front of the judge are extremely convincing that they want to marry this Moroccan partner living abroad, usually in Europe. The media plays a huge role in presenting and instilling the belief in the European dream. It’s difficult for magistrates to turn down their request. In Morocco, everyone wants to leave. The images of the perfect life abroad bear on their impressionable minds and therefore, often, make them ‘impressionable’ to foreigners*.*”* (Participant 2 – academic)

Participants from women’s rights organisations and health care facilities acknowledge the so-called *legal pragmatism* that magistrates are confronted with, yet point out that the social reality paints a whole different picture. Instead, health professionals and women’s advocates in the field believe that judges repeatedly rule in a manner that is inconsistent with the progressive spirit of the law. Whereby appearing to protect the individual, often their patriarchal visions of the family unit motivate their decision [1]. This might be influenced by the fact that the majority of the judges are male and married [27]. Statistics of the Ministry of Justice confirm that only a minority of judges perform the medical and psychological exam or social inquiry into the reasons for marriage, despite this investigation being called for by law^l^.

*“Frequently we observe that the judge contents himself with a personal evaluation based on the physical appearance of the girl. If she looks medically fit for marriage and, from a social point of view, is capable of running a household, the judge agrees to the marriage.” (*Participant 21 – lawyer).

*“We deal with girls as young as 14, which is well below the minimum age stipulated in the Moudawana. Often there is an element of deceit: when girls look much older than their age, the judges don’t blink an eye.”* (Participant 15 – health care worker)

Since the decision to grant an underage marriage cannot be reversed^m^, judges seem to be taking matters firmly into their hands. The only other way to get out of the marriage is to divorce. Whereas this is perfectly possible on paper under the new provisions of the Moudawana, women and girls will often prefer to stay in a forced marriage than divorce. This is due to all the difficulties that the status of divorce brings about.

*“Divorce rarely offers solutions for women. They have limited means to support themselves, let alone their children, and there’s a lot of stigma attached to it.”* (Participant 5 – NGO representative)

*“By calling for and encouraging divorce, feminist militants might do more harm than good.”* (Participant 2 – academic)

Participants pointed out that unmarried women are not considered to have any rights and are abandoned by their families. Unwed mothers, and their children, are among the most legally and socially marginalized people in Morocco [4]. This reality is what women face on a daily basis.

*“It is a very serious issue socially and in the public conscience.”* (Participant 8 – government representative)

Participants from the health care sector also pointed to the perpetuating vicious cycle of forced marriage, spanning several generations.

*“Forced marriage is in itself a risk factor for* [further] *forced marriage. It has a considerable psychological impact of which children are the victims. Especially if the ensuing divorce means that the child must leave school.”* (Participant 17 – health care worker)

*“Deteriorating health in the victims of forced marriage affects not only the children of the marriage, but the (extended) family at large.”* (Participant 9 – NGO representative)

### Impact of legislation

Despite the *revolutionary* spirit of the new Family Code, the application of its provisions is still not presenting sufficient guarantees and protection 8 years after its introduction. Participants alluded to the prevailing patriarchal mentality throughout all layers of society.

*“A woman’s identity has historically been linked to men. New laws or codes will not change that fact from one day to the next.”* (Participant 1 – teacher)

*“Despite the fact that the Family Code (Moudawana) sets forth a collection of articles, these do not go far enough in addressing what is required. It should not set forth any terms of marriage that do not radically tackle the problem of forced and child marriages, rather than doing so in a haphazard fashion.”* (Participant 20 – lawyer)

Nevertheless, several participants underscored the importance of a legal provision, stressing that the constructive far-reaching effects of the new Family Code in the long term cannot be underestimated*.*

*“If you have legal provisions in place and a legal document to fall back on, you can fight for your right and build on it. It’s a base, a foothold.”* (Participant 3 – academic)

Yet, the law is sometimes put aside, due to ignorance or social practices^n^ firmly embedded in the patriarchal structure in pockets of society. The result is an engrained unawareness among girls and women of their rights.

*“In rural areas, many child marriages escape from being included in the Ministry of Justice’ statistics. These marriages take on the form of a simple ‘Fatiha’ (declaration), remain unregistered, and transform girls into married women without them even being aware of it.”* (Participant 9 – NGO representative)

Additionally, the Penal Code criminalizes all sexual relationships, including consensual, outside of marriage. As a result, the topics related to sexuality are social taboos that people avoid approaching, especially with young people [36]. Regarding sexuality, teachers also adopt attitudes engrained in traditional values of decency, characterized by their religious faith, which hampers the spread of knowledge on sexual and reproductive health [37].

*“With sexual relations outside of marriage being considered illegal and an offence, the knowledge on sexual and reproductive health remains substandard.”* (Participant 7 – NGO representative)

*“Because of social stigma, we don’t discuss these things. Many boys and girls do not receive sexual education.”* (Participant 13 – NGO representative)

Alarmingly, among the factors that increase the risk of forced marriage, participants referred to legal provisions. Besides being a compelling instrument to enhance lives, Moroccan law also has the power to harm. Article 475 of the Moroccan Penal Code, spurred on by the illegality of sexual relations outside of marriage, gives free reign to a forced marriage, thus legalizing the crime. When an underage girl is raped, the perpetrator can escape punishment and a prison sentence by marrying the girl. An aggressor used this legal escape route earlier this year, after which Amina, a 16-year old rape victim from Larache, committed suicide in March 2012. The highly mediatised incident resulted in mass protest in front of parliament and court buildings throughout the country [38,39].

*“Rights of women and girls are being raped by unjust Moroccan laws. It’s basically ongoing institutional rape.”* (Participant 12 – NGO representative).

### The role of education

The role of education is paramount. In all of the interviews, reference was made to the importance of knowledge. Adult literacy rate is 56,1% for the general population aged 15 years and older. The overall female adult literacy rate is lower at approximately 43,9%, compared to men at 68.9% [40]. In the younger generation (15–24 years), literacy rates have risen steadily in the past 10 years. Nevertheless, boys and young men in this age group still outrank girls and young women by 86,7% to 72,1% [40]. Boys also outnumber girls in primary net enrolment rates [41].

Educational attainment is an important determining factor of marriage and adolescent childbearing. The number of women in Morocco who have had five years or more of schooling drops dramatically among women who married before the age of 20 [42]. Over 70% of this group only attended school between 0 and 4 years [42].

*“It all comes down to education, in the family from early childhood onwards, the primary level of education, and the defining secondary years of schooling.”* (Participant 1 – teacher)

*“Education is the best prevention for forced marriage and child marriage. But according to a national study, approximately 80% of public schools in the Moroccan countryside have no water or electricity.”* (Participant 8 – government representative)

Education starts at home, with the parents, grandparents and extended family. With illiteracy rates higher among older generations, sensitization and outreach activities about the detrimental effect of a forced child marriage should also be aimed to increase knowledge among girls’ and boys’ relatives. Paternal control issues frequently came up in interviews, stemming the girls’ freedom of expression and forcing then into marriage. It was mentioned that uncles and aunts have the right to intervene with the same authority as that of the father. Several participants emphasized *parental authority* in general, equally drawing attention to mother’s roles in marrying their children off, both for economic and social reasons.

*“We have also dealt with maternal authoritarianism, especially intense among educated mothers. This brings to mind a case I experienced in the city of Tangiers, a mother who had decided to marry off her daughter to the son of a friend and business partner, since she stood to profit from this relationship in the field of business which she ran together with her friend. They were therefore married by force.”* (Participant 20 – lawyer)

“*One of the difficulties in handling forced marriage cases is dealing with mothers. They have an engrained belief in paternal authority and the need for a girl to be married. They are ruled by fear that their daughter might be rejected by society otherwise.”* (Participant 6 – government representative)

A number of participants called for the schools to work in conjunction with the home front, by involving the extended family and *“convincing them that the life of a girl is her property”* (Participant 22 – lawyer). Participants believe schools can offer protection by speaking with parents and helping the girls psychologically. For that aim, several professionals dealing with victims resolutely stated that *“school committees”* should be empowered to conduct outreach activities and to intervene (Participants 15 – health care worker; 18 – teacher; 22 – lawyer).

*Forced marriage and child marriage is an emanation of firmly fixed beliefs in masculine authority. Women, especially in the older generation, would never question the superiority of the husband. Consequently, men have been raised to be number one by their mothers, grandparents, social surroundings, etc. Girls were educated to be nice, docile and subservient. So when women challenge their status, it will initially bring about resistance and sometimes violence.* (Participant 9 – NGO representative)

Participants call for the school system to take gender-based violence into account. Moreover, as a number of participants claimed, gender inequality appears to be embedded in education:

*“Symbols of male power emanate from the King onwards and downwards into our educational system. In our workshops with school children, for example, one of the boys stated «I’m happy not to have sisters, otherwise I’d have to look after them and control them».”* (Participant 8 – government representative)

*“The school system perpetuates the social construction that is thousands of years old where the male figure is superior, even though she might think she’s emancipated. Victimization is still ongoing in all areas: sexual, economical, etc.”* (Participant 2 – academic)

This is corroborated by a study on gender bias in Moroccan schoolbooks, which reviewed textbooks in Morocco for human rights and gender equality [43]. The research, conducted in cooperation with the Moroccan Ministry of National Education, concluded that few female authors were used either in the development of the textbooks or referenced within them^o^. Publishers also tend to select women as textbook authors for subject matters like home economics, thus reaffirming gender stereotypes [43]. Furthermore, teachers are almost exclusively male, especially in rural schools [44].

*“Girls, and boys for that matter, are educated in a system that recognizes male supremacy.”* (Participant 7 – NGO representative)

Initiatives are being taken by NGO’s and women’s associations to organise awareness-raising events, to reinforce capabilities of women and girls, to expose belief systems and self-limiting patterns and to reconstruct their identities based on equality. All this takes time and money. Resources are limited however; so most organisations focus more on assistance as a curative measure, providing a listening ear, legal aid, mediation and health expertise. The importance of including men in the approach was recognized by all of the participants. This need was also identified at government level. One academic participant referred to the recently established ‘Master of Gender and Public Policy’ at the University Mohammed V in Rabat, a course that examines the subject of violence in depth, in order to deconstruct fixed views and explore the factors that lead to gender-based violence in the first place. The study programme, the first of its kind in 2009, is a scheme that was set up in partnership with the Ministry of Solidarity, Woman, Family and Social Development for the advancement of national expertise on the topic of gender equality and for the promotion of gender mainstreaming in the country.

Not only formal education will limit the risk of forced marriage, according to several participants it is up to the media, and especially the television, which figures prominently in households, to bring awareness and sensitization.

*“Nowadays, television is a central part of every home in Morocco. Using this medium constructively, information and knowledge can be spread to members of several generations simultaneously, both male and female alike. Outdated concepts could be obliterated in a short time, with limited resources.”* (Participant 11 – NGO representative)

### The economic factor

Participants from women’s organisations and NGO’s view women as agents of grassroots change. Yet it is repeatedly mentioned that education alone is not enough.

*“Education goes hand in hand with economic development. Only a very encompassing approach will ultimately lead to greater results.”* (Participant 7 – NGO representative)

For example, participants reported that most judges in Family Courts authorize underage marriages for economic reasons, especially if the region is poorer. There was also mention of a strong link between economic development and sexual and reproductive rights.

*“A woman with an income has more power in the relationship. She can exercise her rights to contraception and family planning.”* (Participant 3 – academic)

A compelling tool for women upon entering marriage is the marriage contract. With the new Moudawana, women were granted more rights in the negotiation of marriage contracts.

*“A marriage contract, negotiated before tying the knot, is a good initiative to raise awareness and empower women at a very basic level by educating them on their rights and by safeguarding them economically in the ensuing marriage”.* (Participant 4 – NGO representative)

Participants pointed to the current job market as a compelling barrier for economic advancement and independence. Formal education does not automatically guarantee a job in today’s economy. Therefore, lots of young men and women aspire to immigrate to Europe.

*“The youth have now adopted a new vision as a result of satellite television and the immigration culture, as well as technology*.” (Participant 22 – lawyer)

This, in turn, has an impact on marriage.

*“The marriage market has taken on a transnational element, as many young girls dream of tying the knot with a Moroccan husband living in Europe.”* (Participant 2 – academic)

The overall sentiment among participants was that legislation has advanced, yet the economic development still lags behind. This is especially the case for women, who are more illiterate than men. Economic inequalities also form the basis for less education, perpetuating a vicious cycle and resulting in less awareness about basic sexual and reproductive rights. However, several participants also call for legal amendments to support women’s economic independence seeing that the Moudawana did not eliminate gender discrimination in the inheritance system.

*“Despite far-reaching reforms some years ago, economic discrimination is still part of Moroccan law. This is most notable in the provisions regarding inheritance, which states that a woman receives only half of what her brother receives. This perpetuates the vicious cycle of economic insecurity.”* (Participant 3 – academic)

Favourable factors, such as schooling and legal amendments should have decreased the number of forced and child marriages. Nevertheless, several participants faced a rising amount of instances in the past years, predominantly in more deprived regions, pointing to a possible link between socio-economic hardship and the risk of being forced into wedlock. By marrying off his daughters, fathers can not only lessen the financial burden on the household, but also receive a dowry in exchange, both of which act as an incentive [2,45].

*“Especially in certain areas of the country, notably in the Greater and Lesser Atlas, the phenomenon is growing steadily. Girls are married off after they reach the age of 12 or 13, and these children have absolutely no power over what the families or village notables may or may not decide. The socio-economic status of this group is frequently precarious. This type of marriage will result in problems that even the law is unable to resolve.”* (Participant 20 – lawyer)

## Discussion

The findings indicate that the application of the new Family Code, 8 years after its implementation, still faces a number of barriers and resistance. Proof of this is the staggering number of child marriages that are approved by the courts [46]. Similar to other studies [46-48], our research lays bare an underlying conservative attitude in applying the law, which goes against the very spirit of the Moudawana. Lack of guidelines for those in charge of applying the new Family Code could be partly responsible, given that the law did not guarantee any training of the judiciary in its new provisions [1]. Yet, article 54 of the Moudawana further underscores the exceptional nature of child marriage, stating that all possible measures to guarantee the children’s natural development must be taken, thereby protecting their physical and psychological integrity. Too often are girls considered *’of age according to the Sharia’* by both magistrates and relatives alike [5]^p^. The Moudawana itself might be partly responsible for this trend. The last article provides judges with general guidelines, stating that the judge should turn to the principles of justice and equality under Morocco’s Malekite school of Islam when there is doubt or when the law does not provide an answer [1]^q^. Judges’ personal views of marriage and the family tend to be the grounds for their rulings instead of the law. In response to the situation, the Ministry of Justice adopted the legal system reform program (2009–2012), which, amongst others, aims to achieve a number of 1500 trained judges and ongoing training sessions for magistrates [49]. More specifically geared towards the Family Court magistrates, the American Bar Association Rule of Law Initiative (ABA-ROLI) worked with the Institut Supérieure de la Magistrature (national judicial training center), judges, prosecutors and academics to develop an interactive e-learning curriculum on the Moudawana, which includes units on gender equality under international human rights law and Islam [1].

So, overall, the patriarchal underpinning in Moroccan society seems unlikely to disappear in the near future [46]. The institute of marriage remains a cornerstone of society and is held in such high esteem in the public consciousness that *unmarried women and men are considered a threat to the social order* because their sexuality would risk provoking social chaos [2,4,45,50]. As a result, despite the Moudawana’s provisions, individual women still do not necessarily have freedom from familial and community-based pressures [2].

The recent adoption of the new constitution in July 2011, firmly establishing the principle of equality between men and women for the first time in Moroccan history, raised expectations that there should be no more room for misapplication of the Moudawana’s provisions [3]. The fact that sexual relations outside of marriage are illegal, and abortion is criminalized as a public morality offense, considerably affects people’s attitudes, health and access to their rights^r^. Morocco also does not recognize that violence against women is a public health problem. Therefore it was not included in the priorities of the Ministry of Health’s strategy in 2008, nor in 2010 [47]. The legal prohibition of sexual relations outside of marriage, and the spotlight on virginity or female intactness upon marriage, also limits the spreading of knowledge on sexual and reproductive health [4].

There is seemingly a contradiction in the fact that participants regard education as paramount to prevent forced marriages, yet also point out the fact that forced and child marriages occur in every level of society, regardless of age or educational levels.

This apparent paradox lays bare the difference between theoretical knowledge, often done through disseminating information or awareness raising activities, and empowerment. The latter goes much further and reaches a point when merely knowing about one’s rights becomes a true foothold and tool that is utilized in an effective manner. In several interviews this surfaced as girls’ and women’s self-worth. One NGO representative phrased it as *“her innate confidence to seek solutions, rather than maintain the status quo”.* (Participant 5 – NGO representative) The importance of self-worth cannot be underestimated as a pivotal factor for standing up for one’s rights. It transmutes the victim-paradigm into that of an actor. Often this is linked to economic opportunities as well. By holding a job and earning an income, women’s self-confidence is raised, which, in turn, leads to greater self-worth. Taken as a whole, the factors education and economics must be addressed together, as they are linked in a vicious self-perpetuating circle. Nonetheless, public opinion about women’s place in society still portrays a far from egalitarian society. When considering education, 58,3% of men and 42% of women believe that university-level studies are more important for men than for women [46]. Of all the men and women who were surveyed, 86,9% of both men and women believe that men should be given preference over women when seeking employment [46]. Notwithstanding, Morocco has achieved substantial gains in education in recent years. Major improvements were especially notable among rural girls and women. In the age category 15–21 the proportion of females who had ever attended school reaches 73%, representing a significant increase compared to rural women aged 22–29, where only 40% of this age group had ever seen the inside of a classroom [51]. The crux appears to be at the transition from school to the job market. Women face many more obstacles than men. In a World Bank survey, 30,6% of young women stated that they were unwilling or unable to work because their husbands do not allow it, while another 23,3% were forbidden by their parents. On top of that, another 22,9% reported that they were too busy with household chores to work [51]. Vast numbers of women appear to be discouraged from entering the workforce, making them dependent on male relatives or husbands, thus further hindering them to fully exercise their decision-making power with regards to sexual and reproductive health.

On the whole, seeing that the objective of this research study was to explore factors that compound the problem of child and forced marriage in Morocco, the influence of context on a macro-level scale emerges as paramount. Factors on this level include education, economy and the prevailing policy framework, such as legislation. Of these three factors, the participants repeatedly referred to the new Family Code or Moudawana as having the greatest impact on advancement of women’s rights in the sphere of marriage, yet simultaneously recognizing that recurring resources and attention are needed to ensure that women actually enjoy equality in the family [1]. Adding to its importance, the Moudawana is spilling over into the public sphere, as women’s organisations and civil society continuously shape the public debate around it. As a result, the Penal Code is under review and the new 2011 Constitution now incorporates the principle of equality between men and women. Most importantly, an NGO-drafted bill on violence against women was submitted to the Secrétariat Général du Gouvernment in early 2010 [3,52]. Considering the results of the 2011 national study on violence against women, which revealed that nearly 62,8% of the 9.5 million women surveyed had suffered an act of violence in the preceding 12 months, action cannot be taken soon enough [3]. This same study shows that women who married without consent are almost three times more likely to experience partner violence, including sexual violence [30]. Amina’s suicide in March 2012, after being forcibly married to her rapist, has grasped media attention and put further pressure on the Moroccan government to act.

If all of the above-mentioned proposed measures are implemented properly, with the necessary awareness surrounding the new legislation, it could signal a shift in public perception, effectively condemning the continuation of child and forced marriage. Yet, it remains to be seen how far the government will effectively go, as one participant summarizes the present situation: *“Despite Morocco being recognized as a good example and a “best practice” on women’s rights in the region, lots of issues are not being tackled. Single mothers and their children are discriminated as they are not considered to have rights as a family, and gender-based violence is a serious concern.”* (Participant 8 – government representative)

## Conclusions

The study provided a comprehensive understanding of the factors that compound the problem of child and forced marriage in Morocco. From the viewpoint of professionals, who are closely involved in tackling the issue, policy measures and the law have the greatest potential to bring child marriages and forced marriage to a halt. However, the implementation of new legal tools is facing barriers and resistance. Additionally, the legal and policy framework should go hand in hand with both education and increased economic opportunities. Education and awareness-raising of all ages, in conjunction with the use of popular media, is considered essential, seeing that parents and the extended family play a huge role in marrying off girls and young women. Schools should be empowered to work with both students and the home front.

Overall, preventive action seems to be overlooked in favour of curative measures. Due to lack of funding and resources, women’s associations and NGO’s are only able to offer assistance to those who actively seek help. A large group of potential victims of child and forced marriages is thereby overlooked. In the long run, investing more in prevention would reduce the detrimental effects to victims and families, thereby lessening the burden on society in general, especially health costs and psychological effects on women.

## Endnotes

^a^The new Moroccan Family Code or Moudawana entered into force on February 5, 2004.

^b^Article 19 Moudawana [53]. For boys, the age limit to enter into marriage was always set at 18 years.

^c^Article 20 Moudawana [53].

^d^Requests for underage boys to marry comprise only a tiny fraction of the totality. In 2007 this category represented 0,98% of all official demands [54].

^e^Article 20 Moudawana stipulates that the Family Affairs Judge in charge of marriage may authorize the marriage of a girl or boy below the legal age of marriage, in a well-substantiated decision explaining the interest and reasons justifying the marriage, after having heard the parents of the minor who has not yet reached the age of capacity or his/her legal tutor, with the assistance of medical expertise or after having conducted a social enquiry [53].

^f^In 2010, the approval rate was 92,2% [6].

^g^Article 16(2): “Marriage shall be entered into only with the free and full consent of the intending spouse”.

^h^Art. 1 CRC: *“For the purpose of the present Convention, a child means every human being below the age of eighteen years unless under the law applicable to the child, majority is attained earlier.”* (adopted by the UN General Assembly in 1989). Morocco ratified the Convention on the Rights of the Child (CRC) in 1993.

^i^Article 16(2) CEDAW (adopted by the UN General Assembly in 1979). Morocco ratified the Convention for the Elimination of all forms of Discrimination Against Women (CEDAW) in 1993. In addition, article 21(2) of the African Charter on the Rights and Welfare of the Child (1990) requires states to prohibit child marriage and to adopt legislation “to specify the minimum age of marriage to be 18 years”. However, Morocco is not a member of the African Union and is not a signatory to the African Charter on the Rights and Welfare of the Child.

^j^The thematic framework is arranged according to certain features/characteristics, based on the heuristic model developed by Hooghiemstra, which encompasses structural macro-level factors as well as the role of social networks (meso-level) and personal characteristics (micro-level).

^k^See the ‘Background’ section for more information on the prevailing concepts of forced marriage and child marriage.

^l^Figures demonstrate that of all the requests for underage marriage 31,05% performed a social inquiry (*investigation*), 43,49% carried out a medical and psychological exam (*expertise*), and only 25,47% did both the social inquiry and medical/psychological exam [54].

^m^Article 20 Moudawana further stipulates that *the decree granting the petition to marry for a minor who has not reached the age of legal capacity for marriage is not open to appeal*[53].

^n^Unregistered marriages referred to as ‘Fatiha’ are so named because they are solemnised by recitation of the first or opening chapter (the Fatiha) of the Koran and celebrated in a gathering. The woman is given away by her father, or in his absence by his nearest male relative. A Fatiha marriage is not legally recognized, nor does it fulfil the criteria of a valid marriage set by article 5 of the Moudawana.

^o^At the primary school level, the reference to female authors is limited to 5% in comparison to male authors. This percentage is even lower at the junior high school level, which shows references of only 3% for female authors [43].

^p^The Sharia is Muslim or Islamic law, drawn from the Qu’ran. It is a comprehensive framework of rules, regulating not only the spiritual sphere, but every detail of life. By becoming a member of the Organization of the Islamic Conference in 1969, Morocco is not just a Muslim country in the cultural sense, but is also adherent to Sharia law.

^q^Art. 400 Code de la Famille (‘Moudawana’), Bulletin Officiel No. 5358 (6 Oct. 2005) [53]. The Malekite school is one of the four schools of religious law within Sunni Islam. It is followed by approximately 35% of Muslims, notably in North and West Africa.

^r^See articles 490–491 Code Pénal 1962 and articles 449–458 Code Pénal.

## Competing interests

The study is part of a larger research project funded by the Flemish Interuniversity Council (VLIR) - Institutional University Development Cooperation. The authors assure the absence of competing interests.

## Authors’ contributions

AS contributed to the study design, collection of data, analysis and manuscript writing. MT and EL supervised the study process from design to manuscript editing, and critically reviewed the manuscript for intellectual content. HO participated in the collection of data and analysis. WZ and HK assisted in the data collection. All authors read and approved the final manuscript.

## Pre-publication history

The pre-publication history for this paper can be accessed here:

http://www.biomedcentral.com/1472-698X/13/43/prepub

## References

[B1] Zoglin K Morocco’s Family code: improving equality for women Hum Rights Quart 2009 31 4 964 984 10.1353/hrq.0.0121

[B2] Eisenberg AM Law on the books vs. Law in action: under-enforcement of Morocco’s reformed 2004 family Law, the moudawana Cornell Inter Law J 2011 44 3 693 728

[B3] Human Rights Council Report of the Working Group on the Issue of Discrimination Against Women in law and in Practice 2012 United Nations, New York: Mission to Morocco [A/HRC/20/28/Add.1]

[B4] Bordat SW Kouzzi S Legal Empowerment of Unwed Mothers: Experiences of Moroccan NGO’s 2009 Rome: Legal Empowerment Working Papers International Development Law Program

[B5] Ligue Démocratique pour les Droits des Femmes (LDDF), Centre d'Information et d'Observation des Femmes Marocaines (CIOFEM) Enquête sur les rapports sociaux de genre dans la région de Larache. L'an I du nouveau Code de la Famille. Quel statut socio-juridique pour les femmes? 2006 Casablanca: LDDF-CIOFEM

[B6] Haut-Commissariat au Plan Les indicateurs sociaux du Maroc en 2011 2011 Rabat: Direction de la Statistique http://www.wmaker.net/myhcp2011/downloads/Indicateurs-sociaux_t11880.html

[B7] Salaheddine A Droits de la femme: 41.098 actes de mariage de mineures en 2010 2012 Maroc: Aujourd'hui http://www.aujourdhui.ma/maroc-actualite/societe/droits-de-la-femme-41.098-actes-de-mariage-de-mineures-en-2010-96337.html

[B8] UNICEF Early Marriage: A Harmful Traditional Practice: A Statistical Exploration 2005 New York http://www.unicef.org/publications/files/Early_Marriage_12.lo.pdf

[B9] Gangoli G McCarry M Child marriage or forced marriage? South Asian communities in north east England Child Soc 2009 23 418 429 10.1111/j.1099-0860.2008.00188.x

[B10] Bunting A Stages of development: marriage of girls and teens as an International Human Rights issue Soc Leg Stud 2005 14 1 17 38 10.1177/0964663905049524

[B11] Rude-Antoine E Forced Marriages in Council of Europe Member States. A Comparative Study of Legislation and Political Initiatives 2005 Strasbourg: Directorate General of Human Rights

[B12] Hester M Chantler K Gangoli G Devgon J Sharma S Singleton A Forced Marriage: The Risk Factors and the Effect of Raising the Minimum age for a Sponsor, and of Leave to Enter the UK as a Spouse or Fiancé(e) 2007 London: Home Office

[B13] Nour N Child marriage: a silent health and human rights issue Rev Obstet Gynecol 2009 2 1 51 56 19399295 PMC2672998

[B14] Gangoli G Chantler K Protecting victims of forced marriage: is age a protective factor? Fem Leg Stud 2009 17 267 288 10.1007/s10691-009-9132-7

[B15] Phillips A Dustin M UK initiatives on forced marriage: regulation, dialogue and exit Pol Stud 2004 52 3 531 551 10.1111/j.1467-9248.2004.00494.x

[B16] Anitha S Gill A Coercion, consent and the forced marriage debate in the UK Fem Leg Stud 2009 17 2 165 184 10.1007/s10691-009-9119-4

[B17] Samad Y Eade J Community Perceptions of Forced Marriage 2002 London: FCO (Foreign and Commonwealth Office)

[B18] Jain S Kurz K New Insights on Preventing Child Marriage. A Global Analysis of Factors and Programs 2007 Washington DC: International Center for Research on Women

[B19] Otoo-Oyortey N Pobi S Early Marriage and Poverty. Exploring Links for Policy and Programme Development 2003 London: Forum on Marriage and the Rights of Women and Girls

[B20] Koenig MA Zablotska I Lutalo T Nalugoda F Wagman J Gray R Coerced first intercourse and reproductive health among adolescent women in Rakai Uganda Int Fam Plan Perspect 2004 30 4 156 163 10.1363/3015604 15590381

[B21] Khawaja M Hammoury N Coerced sexual intercourse within marriage: a clinic-based study of pregnant Palestinian refugees in Lebanon J Midwifery Womens Health 2008 53 2 150 154 10.1016/j.jmwh.2007.09.001 18308266

[B22] Nour N Health consequences of child marriage in Africa Emerg Infect Dis 2006 12 11 1644 1649 10.3201/eid1211.060510 17283612 PMC3372345

[B23] Hampton T Child marriage threatens Girls’ health JAMA 2010 304 5 509 510 10.1001/jama.2010.1009 20682925

[B24] UNICEF State of the world’s Children 2011 New York http://www.unicef.org/sowc2011/pdfs/SOWC-2011-Main-Report_EN_02092011.pdf

[B25] UNFPA State of the World Population 2005. The Promise of Equality: Gender Equity, Reproductive Health and the Millennium Development Goals 2005 New York http://www.unfpa.org/swp/2005/pdf/en_swp05.pdf

[B26] Auvert B Buvé A Ferry B Caraël M Morison L Lagarde E Robinson NJ Kahindo M Chege J Rutenberg N Musonda R Laourou M Akam E Study Group on Heterogeneity of HIV Epidemics in African Cities Ecological and individual level analysis of risk factors for HIV infection in four urban populations in sub-Saharan Africa with different levels of HIV infection AIDS 2001 15 Suppl 4 S15 S30 11686462 10.1097/00002030-200108004-00003

[B27] Clark S Early marriage and HIV risk in Sub-Saharan Africa Stud Fam Plann 2004 35 3 149 160 10.1111/j.1728-4465.2004.00019.x 15511059

[B28] Clark S Bruce J Dude A Protecting young women from HIV/AIDS: the case against child and adolescent marriage Int Fam Plan Perspect 2006 32 2 79 88 10.1363/3207906 16837388

[B29] Gangoli G Chantler K Hester M Singleton A Gill A, Anitha S Understanding Forced Marriage: Definitions and Realities Forced Marriage. Introducing a Social Justice and Human Rights Perspective 2011 London: Zed Books 25 45

[B30] Haut-Commissariat au Plan Enquête Nationale sur la Prévalence de la violence à l‘Egard des Femmes 2011 Rabat http://www.hcp.ma/downloads/Violence-a-l-egard-des-femmes_t13077.html

[B31] Ellsberg M Heise L Researching Violence Against Women. A Practical Guide for Researchers and Activists 2005 Washington DC: World Health Organization, PATH

[B32] Menara Mariages de mineures: une souffrance vécue dans le silence 2012 http://www.menara.ma/fr/2012/07/04/63757-mariages-de-mineures-des-victimes-de-violences-sexuelles-puis-conjugales-favorisees-par-les-lacunes-du-code-penal-rapport.html

[B33] Guest G Bunce A Johnson L How many interviews Are enough? an experiment with data saturation and variability Field Methods 2006 18 1 59 82 10.1177/1525822X05279903

[B34] Hooghiemstra E Migrants, partner selection and integration: crossing borders? J Comp Fam Stud 2001 32 4 601 626

[B35] Ritchie J Lewis J Qualitative Research Practice: A Guide for Social Science Students and Researchers 2003 London: Sage

[B36] Siham A Moroccan Agency Supports Sex Education 2011 Magharebia: Rabat

[B37] Sabah S Boujemaa1 A Salah-Eddine K EL Abboudi T Berger D Sexuality education: analysis of Moroccan teachers’ and future teachers’ conceptions US-China Educ Rev 2013 7 8 28 36

[B38] Bruneau C Le Maroc choqué après le suicide d‘une jeune fille violée 2012 Le Figaro http://www.lefigaro.fr/international/2012/03/15/01003-20120315ARTFIG00733-le-maroc-choque-apres-le-suicide-d-une-jeune-fille-violee.php

[B39] Timjerdine F Bennaceri S Suicide de l’adolescente Amina: L’enfance violée face au règne de l’impunité 2012 Aufait Maroc http://www.aufaitmaroc.com/maroc/societe/2012/3/16/suicide-de-ladolescente-amina-lenfance-violee-face-au-regne-de-limpunite

[B40] Haut-Commissariat au Plan Les indicateurs sociaux du Maroc 2010 2010 Direction de la Statistique http://www.wmaker.net/myhcp2011/downloads/Indicateurs-sociaux_t11880.html

[B41] UNICEF At a glance: Morocco statisticshttp://www.unicef.org/infobycountry/morocco_statistics.html

[B42] Yavuz S Changes in Adolescent Childbearing in Morocco, Egypt and Turkey 2010 Calverton MD, USA: Demographic and Health Research Working Papers, United States Agency for International Development

[B43] Human Rights Education Associates (HREA) HREA Presents Results of Study on Gender Bias in Moroccan Schoolbooks 2005 HREA-Maroc, Casablanca http://www.hrea.org/index.php?doc_id=496

[B44] Sadiqi F Azzouzi A Gender Perceptions in Moroccan Culture Cultural and Civilisational Realities 2008 Paris: L’Harmattan 165 189

[B45] Benradi M M’chichi HA Ounnir A Boukaïssi MM Zeidguy R Le Code de la famille. Perceptions et pratique judiciaire 2007 Morocco: Friedrich Ebert Stiftung, Fes

[B46] Desrues T Moreno Nieto J The development of gender equality for Moroccan women - illusion or reality? J Gend Stud 2009 18 1 25 34 10.1080/09589230802584220

[B47] Fédération de la Ligue Démocratique des Droits des Femmes Rapport de la Fédération de la Ligue Démocratique des Droits des Femmes 2012 Maroc: Examen Périodique Universel http://lib.ohchr.org/HRBodies/UPR/Documents/session13/MA/FLDDF_UPR_MAR_S13_2012_LaFederationdelaLigueDemocratiquedesDroitsdesFemmes_F.pdf

[B48] Sadiqi F The Central Role of the Family Law in the Moroccan Feminist Movement British J Mid East Stud 2008 35 3 325 337 10.1080/13530190802525098

[B49] Ministère de l’Economie et des Finances Gender Budget Report. Finance Bill for Fiscal Year 2012 2012 Rabat: Ministère de l'Economie et des Finances

[B50] Flah L ‘Bayrat’ or ‘Spinsters’, Single Women Trapped in Social Stigma 2012 Morocco World News http://www.moroccoworldnews.com/2012/08/52406/bayrat-or-spinsters-single-women-trapped-in-social-stigma/

[B51] World Bank Kingdom of Morocco.Promoting Youth Opportunities and Participation 2012 Washington DC: World Bank

[B52] Global Rights and The Advocates for Human Rights Challenges with addressing domestic violence in compliance with the Convention Against Torture. Joint written statement submitted pursuant to ECOSOC Res. 1996/31, 47th Session of the Committee Against Torture (31 October - 25 November 2011) Morocco http://www.theadvocatesforhumanrights.org/uploads/final_shadow_report_to_cat_re_morocco_response_to_dv_oct_14_2011_sent_to_geneva_2.pdf

[B53] Global Rights English Translation (Unofficial) of the 2004 Moroccan Family Law (Moudawana) 2005 Washington-Rabat http://www.hrea.org/moudawana.html

[B54] Portail Juridique et Judiciaire du Ministère de la Justice et des Libertés du Maroc http://adala.justice.gov.ma/production/statistiques/famille/FR/Mariages%20des%20mineur(e)s.pdf

